# Collagen Matrix vs. Autogenous Connective Tissue Graft for Soft Tissue Augmentation: A Systematic Review and Meta-Analysis

**DOI:** 10.3390/polym13111810

**Published:** 2021-05-31

**Authors:** Cristina Vallecillo, Manuel Toledano-Osorio, Marta Vallecillo-Rivas, Manuel Toledano, Alberto Rodriguez-Archilla, Raquel Osorio

**Affiliations:** 1 Faculty of Dentistry, University of Granada, Colegio Máximo de Cartuja s/n, 18071 Granada, Spain; cvallecillorivas@hotmail.com (C.V.); mvallecillo@correo.ugr.es (M.V.-R.); toledano@ugr.es (M.T.); alberodr@ugr.es (A.R.-A.); rosorio@ugr.es (R.O.); 2 Medicina Clínica y Salud Pública PhD Programme, Faculty of Dentistry, University of Granada, Colegio Máximo de Cartuja s/n, 18071 Granada, Spain

**Keywords:** collagen matrices, keratinized tissue, mucosal thickness, soft tissue graft

## Abstract

Soft tissues have been shown to be critical for the maintenance of both teeth and implants. Currently, regenerative soft tissue techniques propose the use of collagen matrices, which can avoid the drawbacks derived from the obtainment of autogenous tissue graft. A systematic review and meta-analysis were conducted to ascertain the efficacy of collagen matrices (CM) compared to autogenous connective tissue graft (CTG) to improve soft tissue dimensions. An electronic and manual literature searches were performed to identify randomized clinical trials (RCT) or controlled clinical trials (CCT) that compared CTG and CM. Pooled data of width of keratinized tissue (KT) and mucosal thickness (MT) were collected and weighted means were calculated. Heterogeneity was determined using Higgins (I^2^). If I^2^ > 50% a random-effects model was applied. Nineteen studies were included based on the eligibility criteria. When using CTG a higher MT gain (0.32 mm, ranging from 0.49 to 0.16 mm) was obtained than when employing CM. Similar result was obtained for the width of KT gain, that was 0.46 mm higher (ranging from 0.89 to 0.02 mm) when employing CTG. However, it can be stated that, although autogenous CTG achieves higher values, CM are an effective alternative in terms of total width of KT and MT gain.

## 1. Introduction

Nowadays, soft tissue plays a pivotal role in maintaining and improving peri-implant and periodontal health. Adequate dimensions of soft tissue in terms of width of keratinized tissue and thickness of mucosa bring numerous benefits for maintenance, stability and prognosis of both teeth and implants. It has been reported that optimal soft tissue conditions around dental implants can contribute to an improvement in gingival and plaque index, as well as a higher stability of marginal bone in comparison to sites with minimal or lacking keratinized tissue and mucosal thickness [1,2]. Although controversial, the literature has described that a minimum keratinized tissue width of 2 mm is essential for the maintenance of the stability and health of the peri-implant soft tissues [3]. In the case of natural teeth, a poor mucogingival complex can be a predisposition toward localized inflammation resulting in the development of gingival recessions [4] or an apical shift of the gingival margin to the cemento-enamel junction, exposing the root surface. It may cause esthetic complaints, commonly associated with mechanical root wear, hypersensitivity, root caries and poor plaque control [4,5].

For all these reasons, soft tissue augmentation is generating an increasing interest. Soft tissue grafting procedures have been proposed to treat mucogingival defects and achieve both aesthetic and functional results, increasing survival rates of teeth and implants [6]. Major clinical indications could be divided into recession coverage, gain of keratinized tissue, and augmentation of soft tissue volume [7]. Many surgical techniques with different materials to produce soft tissue augmentation in thickness and in width have been described. Regardless of the technique applied, autogenous connective tissue graft (CTG) harvest from the palate is most frequently used [8,9,10,11,12]. Despite the possible benefits of the autogenous tissue graft, there are some crucial disadvantages and limitations. Namely, the morbidity and pain associated with a second operating field [12,13,14], and the limited dimensions of palate donor tissue due to different anatomical factors, covering only a few implants or teeth at one time [13].

To overcome the shortcomings of the autogenous connective tissue, the development of connective tissue substitutes of xenogeneic, allogeneic or synthetic origin, are gaining relevance [15,16]. These biomaterials can reduce the surgical time, diminish the surgical morbidity and increase patients’ acceptance [17]. However, two main criteria need to be fulfilled: good biological behavior permitting modeling and remodeling processes and a volume stability along time [12]. Suitable three-dimensional alternative structures are needed to act as scaffolds that promote cell attachment and migration, providing an appropriate environment for cell proliferation and differentiation. This allows cells to secrete their own extracellular matrix to form a tissue-like organization [18]. Consequently, collagen matrices (CM) have been described as an unlimited alternative to autogenous connective tissue grafting and have been used for soft tissue augmentation around dental implants and for root coverage therapy, showing favorable results [12,19]. Although CM have shown good volume stability allowing sufficient time for cell invasion and new tissue formation, the rapid biodegradation by the enzymatic activity jeopardizes its use as an alternative to autogenous grafting [20,21]. 

In this context, the aim of this systematic review and meta-analysis was to evaluate the evidence related to the efficacy of collagen matrices as an alternative to autogenous connective tissue when used as grafts for soft tissue augmentation, and to compare the clinical success of both surgical procedures.

## 2. Materials and Methods

### 2.1. Study Registration and Protocol Development

Before the execution of the study, this review proposal was registered in the International Prospective Register of Systematic Reviews (PROSPERO) with the identification number CRD42021227177. This systematic review focusing on the efficacy of CM versus CTG for soft tissue augmentation was structured according to the PRISMA-P [22], following the recommendations of the Cochrane Handbook for Systematic Reviews of Interventions [23] and also the PRISMA [24] checklist in order to increase the quality and transparency of the study.

### 2.2. PICO Question and Focused Question

- Population: Patients requiring soft tissue augmentation techniques to augment peri-implant or periodontal keratinized tissue width/thickness for aesthetic purpose and/or functional reasons.
- Intervention: Any type of surgical procedures to augment soft tissue with the application of any type of collagen matrix at peri-implant or periodontal sites.
- Comparison: Autologous connective tissue grafts.
- Outcome: Soft tissue gain (width or thickness) measured in mm [25].

The focused question is: In patients requiring soft tissue augmentation techniques, how effective is the application of a collagen matrix compared to autogenous connective tissue graft in terms of keratinized mucosa height or soft tissue volume gain?

### 2.3. Information Sources and Screening Process

An electronic search with a time filter of 15 years was conducted by two researchers (C.V. and M.V.-R.) covering studies until February 2021 on three online data-bases: The National Library of Medicine (MEDLINE by PubMed), The Cochrane Oral Health Group Trials Register and EMBASE. Only studies published in English, between 2009 and February 2021 were included. An additional hand search was performed identifying previous systematic reviews investigating implant and root coverage procedures for soft-tissue improvements for article identification. Searches were re-run prior to the final analysis in May 2021.Details regarding the search terms are presented in Table 1.

### 2.4. Eligibility Criteria

Studies were selected for inclusion if they met the following criteria: (i) human randomized clinical trials (RCT) or human controlled clinical trials (CCT), (ii) studies dealing with soft-tissue treatments to increase keratinized mucosa or mucosal thickness around teeth or implants, (iii) comparison of connective tissue grafts (control) versus xenogeneic collagen matrices (test), (iv) follow-up of at least 3 months, (v) reported outcome measures of keratinized mucosa or mucosal thickness gain following the surgical intervention. On the other hand, the exclusion criteria were: (i) in vitro and pre-clinical studies, cohort studies, case-control studies, case series, case reports, systematic reviews, (ii) full-text publications not available in English language, (iii) studies with a less than 10 patients, (iv) surgical treatment including materials for guide bone regeneration*,* (v) graft as a material for socket preservation.

### 2.5. Study Selection and Data Extraction 

Two authors (C.V. and M.V-R.) independently screened the titles derived from the online search based on the inclusion criteria. Disagreements were solved by discussion. In the case where a disagreement persists, a third reviewer (R.O.) was decisive and led to an agreement. Cohen’s Kappa-coefficient was calculated as a measure of agreement between the two readers. The final selection based on inclusion/exclusion criteria was made for the full-text articles. For this purpose, all data were extracted independently by two reviewers (C.V. and M.V-R.). Information on the following parameters was acquired as follows: author(s), year of publication, study design, number of patients, age range, dropouts, mean follow-up and range, width of keratinized tissue (KT), mucosal thickness (MT), periodontal parameters, patient-reported outcomes measures (PROMs), pink esthetic score (PES) and complications.

### 2.6. Assessment of Risk of Bias 

The assessment of the risk of bias for the included randomized clinical trials was performed using Cochrane Handbook for Systematic Reviews of Interventions [23]. With Cochrane Collaboration’s tool, each study was analyzed in relation to seven domains (sequence generation, allocation concealment, blinding of the outcome assessor, blinding of participants and personnel, incomplete outcome data, selective outcome reporting and other bias) and categorized as low, medium or high risk of bias when they met all, all but one, or all but two or more criteria, respectively.

For the included non-randomized studies of interventions, a tool called ROBINS-I was used [26]. With ROBINS-I tool, risk of bias was assessed within specified bias domains (bias due to confounding, bias in selection of participants into the study, bias in classification of interventions, bias due to deviations from intended interventions, bias due to missing data, bias in measurement of outcomes, bias in selection of the reported result) and categories for risk of bias judgements were “Low risk”, “Moderate risk”, “Serious risk” and “Critical risk” of bias.

### 2.7. Statistical Analysis 

Descriptive statistics were used to present the primary outcome: efficacy of collagen matrix in terms of gingival thickness gain (mm) and/or changes of keratinized tissue (mm). Weighted means (CI 95%) were calculated, including total sample size, inverse variance and standard error of the treatment effect. Heterogeneity was determined using Higgins (I^2^). If I^2^ > 50% a random-effects models were applied. Statistical significance was set at 0.05. Data were analyzed with RevMan 5.4 (The Cochrane Collaboration, Oxford, UK). Funnel plot was produced by MedCalc 18.2.1 (MedCalc Software Ltd. Ostend, Belgium) to represent systematic heterogeneity.

## 3. Results 

### 3.1. Study Selection 

**Figure 1:**
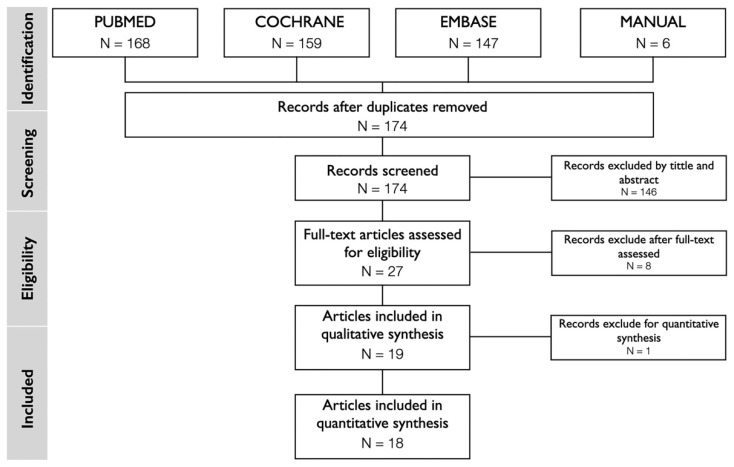
PRISMA flow diagram for studies inclusion process.

Search results based on the PRISMA guidelines are presented in Figure 1. The electronic search provided a total of 474 articles supplemented by a manual search getting 6 more articles [5,27,28,29,30,31]. After duplicates removal, a total of 174 studies were selected for screening of title and abstract. Twenty-eight articles were considered for full-text screening. Nine articles were excluded after careful reading, since they did not meet the eligibility criteria. Finally, 19 studies [12,16,19,27,28,29,31,32,33,34,35,36,37,38,39,40,41,42,43] were included in the systematic review (SR) and one of them was excluded for the quantitative analysis, so the meta-analysis is based on 18 articles [12,16,19,27,28,29,31,32,33,34,35,36,37,38,39,40,41,42]. The reasons for exclusion are reported in Table 2. The inter-reviewer agreement in the screening and inclusion process corresponded to 0.87, and 0.95 with de Cohen’s Kappa for assessment of the title and abstract, and full-text evaluation, respectively.

### 3.2. Study Characteristics 

**Figure 2:**
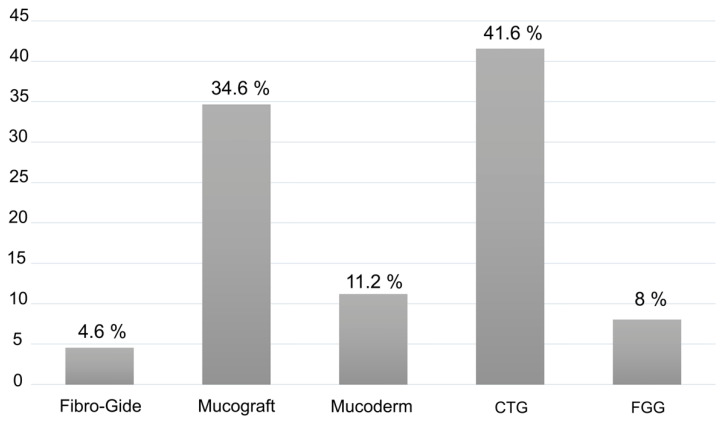
Different therapeutic options for soft tissue augmentation. Data are presented as a percentage of patients treated with each alternative compared to the total number of patients. Connective tissue graft (CTG), free gingival graft (FGG).

Varied applications for soft tissue augmentation were described in the included studies: 12 articles in relation to implant sites [12,16,19,29,31,32,34,37,38,39,42,43] and 7 concerning teeth [27,28,33,35,36,40,41]. All the included studies were RCTs except for three CCTs [31,37,42], but all of them included at least two parallel arms, the use of CTG versus CM. The different therapeutic options used for soft tissue augmentation found in the included studies are shown in Figure 2. The main characteristics for the selected trials are summarized in Table 3, and Table 4 reports on the assessment of soft tissue augmentation used in each study, as well as the primary outcome data.

### 3.3. Quality Assessment of the Included Studies 

**Figure 3:**
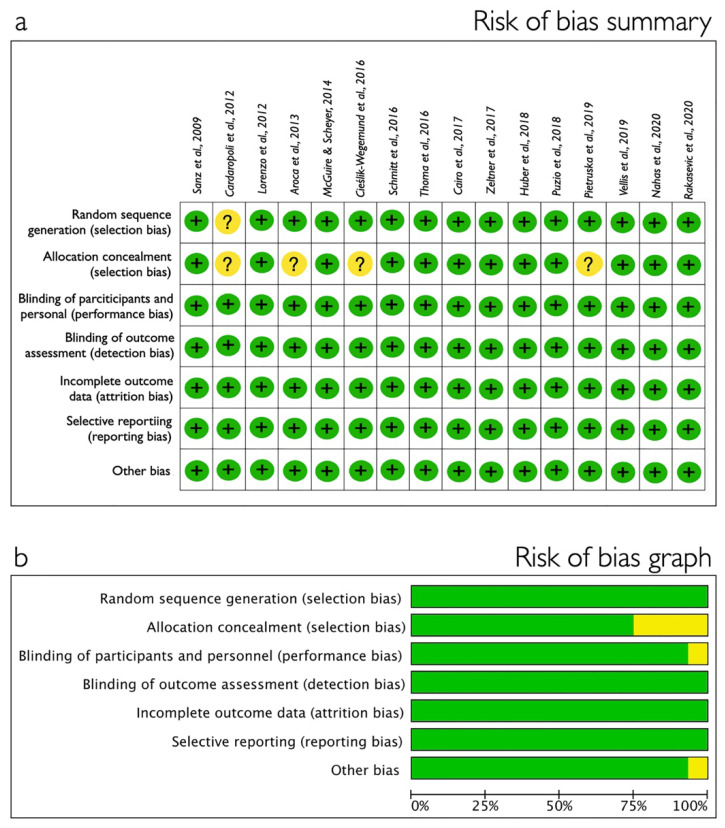
Randomized clinical trial quality assessment using the Cochrane Handbook for Systematic Reviews of Interventions [23]. (**a**) Risk of bias summary: studies were considered as having high (red); moderate (yellow) or low (green) risk of bias. (**b**) Risk of bias graph: each risk of bias item presented as percentages across all included studies.

**Figure 4:**
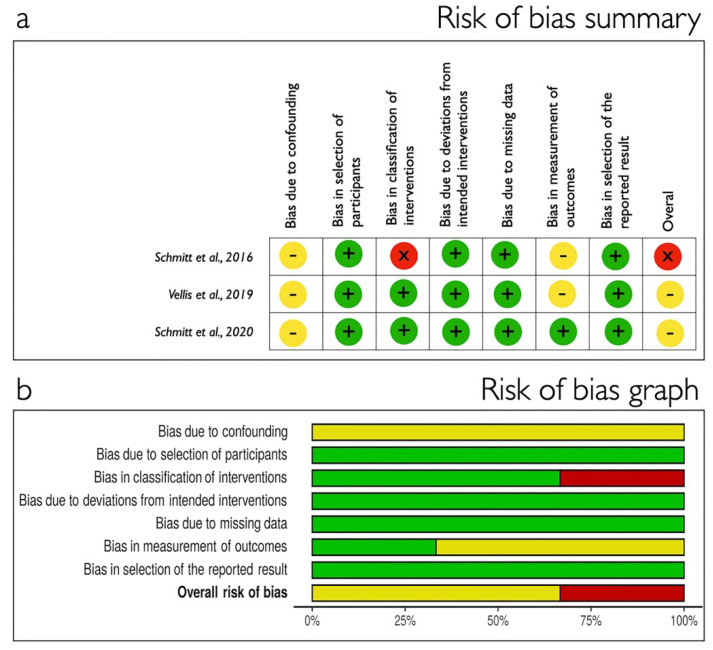
Non-randomized clinical trial quality assessment using ROBINS-I tool [26]. (**a**) Risk of bias summary: studies were considered as having serious (red); moderate (yellow) or low (green) risk of bias. (**b**) Risk of bias graph each risk of bias item presented as percentages across all included studies.

The result of the bias risk assessment for the included papers is reported in Figure 3 for RCT using Cochrane Collaboration’s tool and in Figure 4 for non-RCT in which ROBINS-I tool was used. Most of the RCTs received low risk of bias while the CCTs were classified as moderate or serious risk.

### 3.4. Primary and Secondary Outcomes

**Figure 5:**
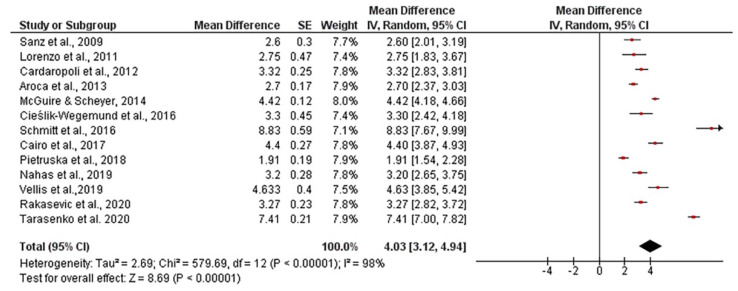
Forest plot for keratinized mucosa width when using autogenous connective tissue. Weighted mean is presented at CI 95%. Heterogeneity was determined using Higgins (I^2^). A random-effects model was applied. Statistical significance was *p* < 0.001.

**Figure 6:**
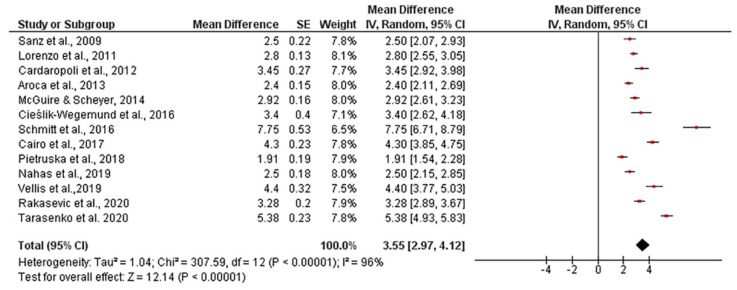
Forest plot for keratinized mucosa width when collagen matrix was used. Weighted mean is presented at CI 95%. Heterogeneity was determined using Higgins (I^2^). A random-effects model was applied. Statistical significance was *p* < 0.001.

**Figure 7:**
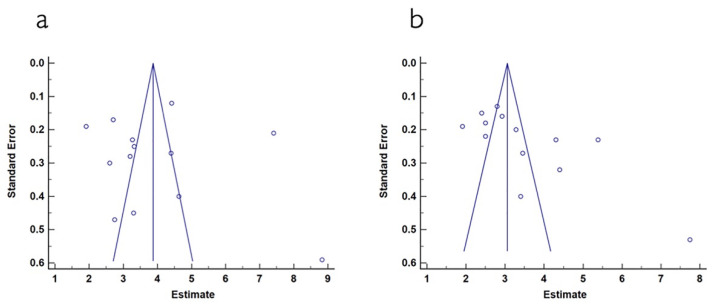
(**a**) Funnel plot for keratinized mucosa width when using autogenous connective tissue. The estimated keratinized mucosa width measurement is on the horizontal axis and study precision (standard error) appears on the vertical axis. (**b**) Funnel plot for keratinized mucosa width in studies using collagen matrix. The estimated keratinized mucosa width measurement is on the horizontal axis and study precision (standard error) appears on the vertical axis.

**Figure 8:**
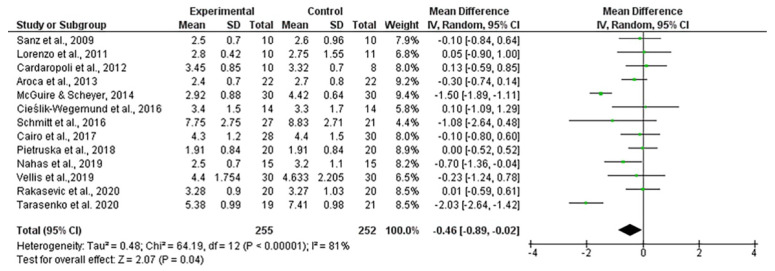
Forest plot for keratinized mucosa width gain. Test (collagen matrix) vs. control (autogenous connective tissue) groups. The weighted mean is presented at CI 95%. Heterogeneity was determined using Higgins (I*^2^*). A random-effects model was applied. Statistical significance was *p* = 0.04.

The mean width of keratinized tissue gain, when using autogenous connective tissue was 4.03 mm, ranging from 3.12 to 4.94 mm (CI 95%). Heterogeneity was I^2^ = 98% (CI 95%) and significance of the random-effects model was *p* < 0.001. When collagen matrix was employed, the mean width gain was 3.55 mm, ranging from 2.97 to 4.12 mm (CI 95%), heterogeneity was I^2^ = 96% (CI 95%) and significance of the random-effects model was *p* < 0.001. Both forest plot graphs of width of keratinized tissue are displayed in Figure 5 and Figure 6. Systematic heterogeneity is displayed at the funnel plot graphs (Figure 7). When comparing test to control groups in terms of width of keratinized tissue, the mean width gain was 0.62 mm higher (ranging from 1.09 to 0.15 mm, CI 95%) when using autogenous connective tissue in comparison to after employing collagen matrix. Heterogeneity is I^2^ = 83% (CI 95%) and significance of the random-effects model was *p* < 0.001 (Figure 8).

**Figure 9:**
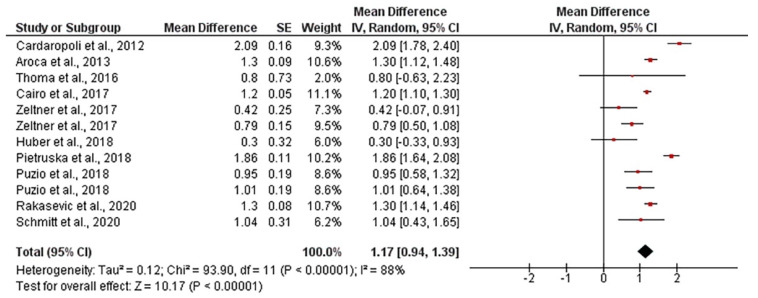
Forest plot for gingival thickness gain when autogenous connective tissue was used. The weighted mean is presented at CI 95%. Heterogeneity was determined using Higgins (I^2^). A random-effects model was applied. Statistical significance was *p* < 0.001.

**Figure 10:**
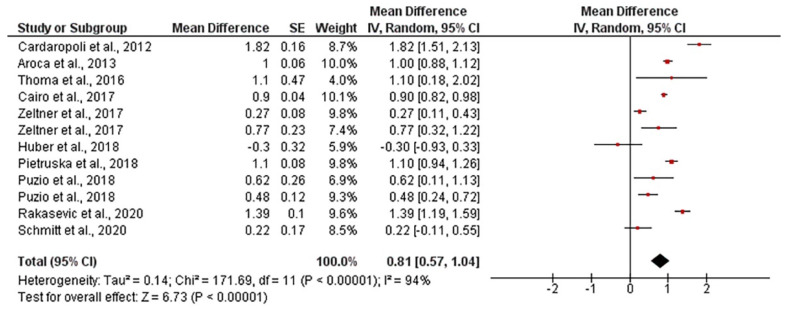
Forest plot for gingival thickness in test group (collagen matrix). The weighted mean is presented at CI 95%. Heterogeneity was determined using Higgins (I^2^). A random-effects model was applied. Statistical significance was set at 0.05.

**Figure 11:**
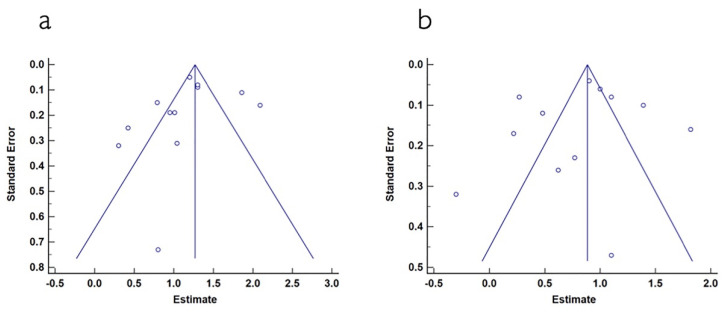
(**a**) Funnel plot for gingival thickness in control group (autogenous connective tissue). The estimated gingival thickness measurement is on the horizontal axis and the study precision (standard error) appears on the vertical axis. (**b**) Funnel plot for gingival thickness in the test group (collagen matrix). The estimated gingival thickness measurement is on the horizontal axis and the study precision (standard error) appears on the vertical axis.

**Figure 12:**
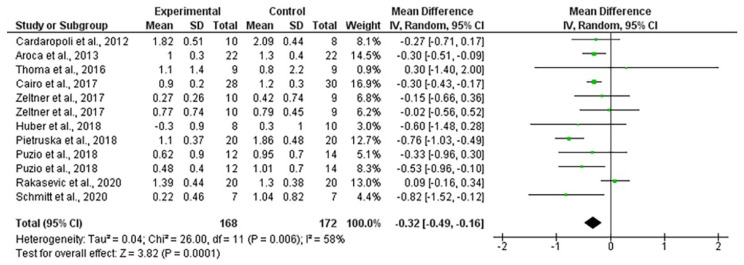
Forest plot for gingival thickness. Test (collagen matrix) vs. control (autogenous connective tissue) groups. The weighted mean is presented at CI 95%. Heterogeneity was determined using Higgins (I^2^). A random-effects model was applied. Statistical significance was *p* < 0.001.

The mean gingival thickness gain when using autogenous connective tissue was 1.17 mm, ranging from 0.94 to 1.39 mm (CI 95%). Heterogeneity was I^2^ = 88% (CI 95%) and significance of the random-effects model was *p* < 0.001. After employing collagen matrixes, the mean gingival thickness gain was 0.81 mm, ranging from 0.57 to 1.04 mm (CI 95%). Heterogeneity was I^2^ = 94% (CI 95%) and significance of the random-effects model was *p* < 0.001. Both forest plot graphs of gingival are displayed in Figure 9 and Figure 10. Systematic heterogeneity is displayed at the funnel plot graphs (Figure 11). When comparing the test to the control groups, the mean gingival thickness gain was 0.32 mm (ranging from 0.49 to 0.16 mm, CI 95%) higher when using autogenous connective tissue than when employing collagen matrixes. The heterogeneity was I^2^ = 58% and the significance of the random-effects model was *p* < 0.001 (Figure 12).

## 4. Discussion 

The aim of this systematic review and meta-analysis was to evidence the efficacy of collagen matrices as an alternative to autogenous connective tissue graft for soft tissue augmentation. The establishment of tight eligibility criteria resulted in a limited number of included studies: three CTs and 16 RCTs. However, it diminishes the risk of bias and strengthens the systematic review [49]. All the included studies have at least two parallel arms: the use of collagen matrix versus autogenous connective tissue graft, and the evaluation of its effectiveness in terms of mucosal thickness and/or width of keratinized mucosa gain. These clinical outcomes were selected since they are the only objective parameters which made it possible to make inter-studies comparisons [1,2,50,51]. A total of 411 patients which undergone soft tissue augmentation surgery, were analyzed. 11 studies evaluated gingival thickness [12,16,19,27,28,33,38,39,41,42,43] and 15 reported data for width of keratinized mucosa [19,27,28,29,31,32,33,34,35,36,37,38,40,41,43]. Among all the included studies, the xenogeneic collagen matrix Mucograft (Geistlich Pharma AG, Wolhusen, Switzerland) is the soft tissue substitute used the most [16,27,29,31,32,33,34,35,37,38,40]. Volume-stable collagen matrix (Fibro-Gide, Geistlich Pharma AG, Wolhusen, Switzerland) [12,19,39,43] was used in four articles. Mucoderm (Botiss biomaterials GmbH, Zossen, Germany), which is also a xenogeneic collagen matrix, was used in four other articles included [28,36,41,42]. 

After performing the systematic review and meta-analysis, it can be inferred that even when a high heterogeneity was attained (I^2^ > 50%), all the random-effects models were highly significant (*p* < 0.001) enough to arise differences between the groups and make it able to state that connective tissue graft is more effective than collagen matrices for soft tissues augmentation around both teeth and implants.

When connective tissue graft was used, a significant gain in gingival thickness and keratinized mucosa were obtained: 1.17 and 4.03 mm, respectively. This increase in the quality of the supportive soft tissues was significantly higher than the one obtained when collagen matrices were used, being 0.81 mm the gained gingival thickness and 3.55 mm the augmentation of the keratinized mucosa. When comparing both groups in terms of width of keratinized tissue, the mean width gain was 0.62 mm higher (ranging from 1.09 to 0.15 mm) when using autogenous connective tissue in comparison to after employing collagen matrix, gingival thickness was also higher in a range of 0.49–0.16 mm. In contrast to these results, Gargallo-Albiol et al. [2] stated that gingival thickness gain was similar (*p* = 0.3) when using collagen matrix or autogenous connective tissue. Other previous systematic reviews and meta-analyses also did not find significant difference between both treatments [2,3,52]. Moraschini et al. [17] and Gargallo-Albiol et al. [2] also concluded that the gain of keratinized mucosa width was similar (*p* = 0.14 and *p* = 0.62, respectively), when comparing connective tissue graft with collagen matrix. These results were probably due to the quite small number of manuscripts finally included in the mentioned systematic reviews (11 and 7 articles, respectively), that did not account for the scientific evidence. Carvalho et al. [52], in accordance with our results, disclosed that the use of connective tissue graft significantly increased keratinized mucosa width when applied to recessions ≥ 2 mm, but as the results were expressed in terms of complete root coverage, then it is not possible to ascertain the real gain using the two different surgical approaches. Therefore, although there are many reviews that analyze soft tissue augmentation in different clinical situations, this is the first study in which the quantitative differences in keratinized mucosa width and gingival thickness are calculated for collagen matrix and autogenous connective tissue, establishing a significant differential gain between each other regardless of whether the recipient is an implant or tooth.

However, the most important point is to be aware of the odds and the limitations of the studied techniques, as a balance needs to be made between the expected improvement of soft tissue dimensions and the drawbacks related to palatal harvesting with the use of autogenous connective tissue graft. It is well known that an adequate gingival thickness plays a crucial role in maintaining periodontal and peri-implant health [53]. So, this gain in soft tissue quality involves the achievement of an improvement in the aesthetic result and a better long-term prognosis of both teeth and implants. Although there is no consensus about which should be the minimal dimensions of soft tissues, it is considered that an adequate amount of keratinized tissue would be 2 mm [3,11,12]. Taking this fact into account and the results encountered by this systematic review and meta-analysis, it may be positive to design a decision tree that helped clinicians to elucidate which of the studied techniques would fit each clinical situation. The requirement of a second surgical site as a donor area increases the morbidity of this procedure, augmenting procedure time [12], post-operative discomfort and complications like bleeding, necrosis and hypo- or anaesthesia [20]. These shortcomings can affect the patient perception of the treatment and—as a matter of fact—the use of a non-autogenous graft avoids these inconveniences and permits a less invasive procedure that provides a faster and more tolerable post-operatory period. All in all, it really makes collagen matrix a valid alternative in some cases.

Although autogenous graft received the highest values in terms of gingival thickness or keratinized mucosa width, studies also assessed, as secondary outcomes, the morbidity after soft tissue augmentation surgery, showing a preference for avoiding the requirement of a second surgical site. This donor area seems to be the major triggering cause of postoperative discomfort. It was concluded by Cairo et al. [38] that the use of collagen matrix resulted in a shorter surgical time to perform the soft tissue augmentation, a reduction of analgesic consumption and a higher final patient satisfaction. In the same line, Sanz et al. [32] and Lorenzo et al. [34] established that the patients in test group (CM) perceived less pain and needed fewer anti-inflammatory drugs. In addition, 30 days after surgery, while the patients that were treated with collagen matrices did not present pain, the patients who received autografts still presented “minor pain”. Differing In contrast to the previously mentioned studies, it was stated by Thoma et al. [12] that although collagen matrix was the best rated, there were no statistical differences (*p* > 0.05) in patient-reported outcome measures (PROMs). Among all the included studies, only Cieślik-Wegemund et al. [36] published data against the collagen matrix in terms of pain, finding significantly greater pain in patients treated with collagen matrix. Another important factor to take into account, but infrequently evaluated across studies, is the integration of the graft in adjacent soft tissues for esthetics evaluation. When assessing this variable, good results were found in both groups [19,32,34,36,38,40,41]. These studies stated that favorable results were obtained in both groups and that when there was a blinded evaluator, both procedures were not able to be distinguished in terms of color or esthetics outcomes. This is contrast to when free gingival graft (FGG) is used. In this case, the collagen matrix shows the best results, with [31,37] reporting that one of the drawbacks of FGG is the discrepancy in tissue color between the graft and the surrounding tissue. Regarding periodontal parameters, no study included in this review found significant differences between groups, establishing a remarkable improvement in periodontal parameters such as probing depth (PD), bleeding on probing (BOP) and clinical attachment level in both study groups.

The main limitation of this systematic review and meta-analysis is the high heterogeneity, probably due to the relatively small sample sizes of the several included studies, which have an average of about 24 patients. It could also be related to the different surgical approaches, operators’ ability, outcomes measured, and data reported. It is encouraged for future researchers to perform more RCTs following the CONSORT guideline and evaluating collagen matrices with higher sample sizes and follow-up time, including patient’s assessment of the technique, postoperative period and aesthetics evaluation. Additionally, standardization the measurement tools would facilitate data extraction and could result in more conclusive outcomes.

## 5. Conclusions

The findings from the present systematic review and meta-analysis suggest that collagen matrix is not as effective as connective tissue graft for soft tissue augmentation, when considering both keratinized mucosa width and gingival thickness. However, collagen matrices also achieve gain values that may be considered as clinically relevant, resulting as a valid alternative for cases where the autogenous connective tissue graft may not be considered as an option, due to patient morbidity.

## Figures and Tables

**Table 1:** Electronic databases and search strategies.

Databases	Keywords
PUBMED	#1 “collagen matrix”[Title/Abstract] OR “extracellular membrane”[Title/Abstract] OR “extracellular matrix”[Title/Abstract] OR “xenogenic collagen matrix”[Title/Abstract] OR “acellular dermal matrix”[Title/Abstract] OR “porcine collagen matrix”[Title/Abstract] OR “porcine collagen matrices”[Title/Abstract] OR “porcine derived collagen matrix”[Title/Abstract] OR “porcine derived acellular dermal matrix”[Title/Abstract] OR “mucograft”[Title/Abstract] OR “mucoderm”[Title/Abstract]” OR “volume-stable collagen matrix”[Title/Abstract] OR “dermal substitute”[Title/Abstract] #2 “soft tissue correction”[Title/Abstract] OR “soft tissue augmentation”[Title/Abstract] OR “soft tissue transplantation”[Title/Abstract] OR “soft tissue graft”[Title/Abstract] OR “guided tissue regeneration”[Title/Abstract] #1 AND #2
Cochrane Library (CENTRAL)	#1 collagen matrix OR extracellular membrane OR extracellular matrix OR xenogenic collagen matrix OR acellular dermal matrix OR porcine collagen matrix OR porcine collagen matrices OR porcine derived collagen matrix OR porcine derived acellular dermal matrix OR mucograft OR mucoderm OR volume-stable collagen matrix OR dermal substitute #2 soft tissue correction OR soft tissue augmentation OR soft tissue transplantation OR soft tissue graft OR guided tissue regeneration #1 AND #2
EMBASE	#1 “collagen matrix” OR “extracellular membrane” OR “extracellular matrix” OR “xenogenic collagen matrix” OR “acellular dermal matrix” OR “porcine collagen matrix” OR “porcine collagen matrices” OR “porcine derived collagen matrix” OR “porcine derived acellular dermal matrix” OR “mucograft” OR “mucoderm” OR “volume-stable collagen matrix” OR “dermal substitute” #2 “soft tissue correction” OR “soft tissue augmentation” OR “soft tissue transplantation” OR “soft tissue graft” OR “guided tissue regeneration” #1 AND #2

**Table 2:** Excluded studies for qualitative and quantitative synthesis with reasons.

Stage	Reason for Exclusion	Articles
Qualitative synthesis	No control group	Ozturan et al. [44]; Eeckhout et al. [45]; Ghanaati et al. [46]
	No valid outcome for this SR	Zafiropoulos et al. [47]; McGuire y Secheyer [48]; Tonetti et al. [5]; Zuiderveld et al. [30]
	Use of the same data of a previous study	Puzio et al. [13]
Quantitative synthesis	Increase the follow-up of a previous study and the follow-up period not considered for meta-analysis	Thoma et al. [43]

**Table 3:** General overview of included studies’ design.

Author	Study Design	Follow-up	Patients	Inclusion Criteria	Outcome Measurements
Sanz et al., 2009 [32]	RCT	6 months	20 patients	Age > 18 years Systematically healthy FMPS < 20% Presenting at least one location with minimal or no KT (1 mm).	KMW, PD, CAL, GI, PI, pain, PAS Changes in KMW
Lorenzo et al., 2011 [34]	RCT	6 months	24 patients	Age >18 years Systematically healthy FMPS < 20% Presenting at least one implant with minimal or no KT (1 mm).	KMW, GI, PI, PD, CAL Changes in KMW
Cardaropoli et al., 2012 [33]	RCT	12 months	18 patients	Age > 18 years No pregnancy or breast feeding Systematically healthy Non-smokers At least two single-rooted teeth with gingival recessions Miller class I and/or II	GR, GT, PD, CAL, KMW Changes KMW
Aroca et al., 2013 [27]	RCT split-mouth	12 months	22 patients	Age > 18 years Systematically healthy Healthy or treated periodontal conditions Presence of at least 3 adjacent Miller class I and II GR on both sides of the maxillary or mandibular arch with an apico-coronal extension (i.e., RD) > 2 mm FMPS < 25%	GRD, GRW, KMW, GT, PD, CAL, PROMs
McGuire and Scheyer 2014 [35]	RCT split-mouth	6 months	30 patients	Age > 18 years No pregnancy or breast feeding Systematically healthy Non-smokers	PS, BOP, RD, KMW, VD, CAL, RMP, IS, Esthetics, Histological
Cieślik-Wegemund et al., 2016 [36]	RCT	6 months	28 patients	Systematically healthy Non-smokers Absence of clinical signs of active periodontal disease An identifiable CEJ Minimum of two adjacent GR of Miller Class I or II on both sides with at least 2 mm in RD API ≤ 15% and SBI ≤ 10%	GRD, GRW, KMW, CAL, CEJ-MGJ, PD, RA
Schimitt et al., 2016 [37]	RCT	60 months	48 patients	Age >18 years Healthy periodontally and systemically Good plaque control. No smokers	KMW, Appearance: color, texture
Thoma et al., 2016 [12]	RCT	3 months	20 patients	Age > 18 years Implant placement at least 6 weeks and maximum 6 months prior enrolment Necessity of STA in a single tooth Two teeth adjacent at each side of the defect with a mean BOP of < 30% BPE < 2	GT, BPE, PI, KMW, BOP, PD, RD, PROMs Volumetric changes of GT
Cairo et al., 2017 [38]	RCT	6 months	60 patients	No systemic diseases or pregnancy. Smokers ≤ 10 cigarettes/day. No probing depths ≥ 5 mm FMPS and FMBS ≤ 15% Need of STA for aesthetic and/or functional reasons in a single-tooth gap at upper and lower jaw No previous STA procedure at experimental site	KMW, GT, BL, RD, PD, BOP, PI, PROMs
Zeltner et al., 2017 [39]	RCT	3 months	20 patients	Age > 18 years Implant placement at least 6 weeks and maximum 6 months prior enrolment Necessity of STA in a single tooth Two teeth adjacent at each side of the defect with a mean BOP of < 30% BPE < 2	Volumetric changes of GT
Huber et al., 2018 [19]	Follow-up of RCT	12 months	19 patients	Age > 18 years Necessity of STA in a single tooth Two teeth adjacent at each side of the defect with a mean BOP of < 30% BPE < 2 Final restoration inserted at implant site	GT, BPE, PI, KMW, BOP, PD, RD, PES, PROMs Volumetric changes of GT
Pietruska et al., 2018 [28]	RCT split-mouth	12 months	20 patients	No pregnancy or breast feeding Systematically healthy, Non-smokers At least two single-rooted teeth with GR Miller class I and/or II ≥ 1 mm deep without loss of CAL FMPS and FMBOP < 20% No active periodontal disease Detectable CEJ No caries lesions or restorations in the cervical area.	GR, GRW, PD, CAL, KMW, GT, FMPS, FMBOP Changes KMW
Puzio et al.,2018 [16]	RCT	12 months	22 patients	Missing single or double teeth in the anterior area of their upper or lower jaw with a proper inter arch relationship with a ridge width (bucco-lingual) greater than 5 mm at its narrowest point and a minimum height of KM of 2 mm measured buccally with a periodontal probe.	GT, gingival biotype
Nahas et al., 2019 [40]	RCT split-mouth	12 months	15 patients	Systemically and periodontally healthy PI: ≤ 20% Multiple bilateral Class I Miller GR, involving canines and premolars (2–3 teeth) with at least one GR ≥ 3 mm Identifiable CEJ At least 1 mm KT apical to the GR	GRD, PI, BOP, PD, CAL, KMW, PROMs Changes KMW
Vellis et al.,2019 [31]	RCT split-mouth	6 months	30 patients	Age > 18 years Systematically healthy No pregnancy or breast feeding	KMW, PD, pain Changes in KMW
Rakasevic et al., 2020 [41]	RCT split-mouth	12 months	20 patients	Age > 18 years Non-smokers and light smokers (<10 cigarettes per day). Systemically and periodontally healthy FMPS < 20% and FMBOP < 20% +1 adjacent Type 1 GRs in both quadrants of the maxillary or mandibular arch with a GR depth ≥ 2 mm Identifiable CEJ Absence of the radiographic signs of periapical infection on the teeth to be treated or on the adjacent teeth.	GRD, GRW, KMW, GT, PD, CAL, HI, RES Changes KMW
Schmitt et al., 2020 [42]	CCT	6 months	14 patients	Age > 18 years No pregnancy or breast feeding Systematically healthy Periodontally healthy (no PD > 4 mm) Situation after early implant insertion and GBR at least 4 months and a maximum of 6 months prior to enrollment.	Volumetric changes of GT
Tarasenko et al., 2020 [29]	RCT	6 months	40 patients	Age >18 years Systematically healthy (ASA I-II) Non-smokers and light smokers (<10 cigarettes per day). Previous placement of one or more implants in the mandibles (3 to 6 months before the beginning of the investigation) without having yet undergone stage-two surgery FMPS and FMBS ≤ 20%	KMW, Inflammation, PROMs, Histology Changes in KMW
Thoma et al., 2020 [43]	Follow-up of RCT	36 months	18 patients	Age > 18 years Necessity of STA in a single tooth Two teeth adjacent at each side of the defect with a mean BOP of < 30% BPE < 2 Final restoration inserted at implant site	GT, BPE, PI, KMW, BOP, PD, RD, PES, PROMs, MBL Volumetric changes of GT

RCT: randomized clinical trial, FMPS: full-mouth plaque score, FMBS: full-mouth bleeding score, STA: soft tissue augmentation, KMW: keratinized mucosa width, GT: gingival thickness, BL: bono Level, RD: recession depth, PD: probing depth, BOP: bleeding on probing, PI: plaque index, PROMs: patient-reported outcomes measures, GI: gingival index, CAF: coronally advanced flap, BPE: basic periodontal examination, PES: pink esthetic score, MBL: marginal bone loss, CCT: controlled clinical trial, CAL: clinical attachment levels, PAS: participants’ aesthetic satisfaction, MBML: mid-buccal mucosal level, IML: inter-proximal mucosal levels.

**Table 4:** General description of the soft tissue augmentation procedures performed in each included study.

Author	STA/ Surgical Technique	XMC Used	Site of Treatments	Time of Grafting	Summary Results
Sanz et al., 2009 [32]	CG: CTG (n = 10) TG: XCM (n = 10) CAF	Mucograft^® a^	Maxilla and mandible	After crown placement	KW: CTG > CM
Lorenzo et al., 2011 [34]	CG: CTG (n = 12) TG: XCM (n = 12) CAF	Mucograft^®^	Maxilla and mandible	After crown placement	KW: CTG < CM
Cardaropoli et al., 2012 [33]	CG: CTG (n = 8) TG: XCM (n = 10) CAF	Mucograft^®^	Maxilla and mandible 22 GR	NR	KW: CTG > CM * GT: CTG > CM *
Aroca et al., 2013 [27]	CG: CTG (n = 22) TG: XCM (n = 22) MCAT	Mucograft^®^	Maxilla and mandible	NR	KW: CTG > CM GT: CTG > CM *
McGuire and Scheyer 2014 [35]	CG: FGG (n = 30) TG: CM (n = 30)	Mucograft^®^	Maxilla and mandible	NR	KW: FGG > CM *
Cieślik-Wegemund et al., 2016 [36]	CG: CTG (n = 14) TG: XCM (n = 14) TT	Mucoderm^® b^	Maxilla and mandible CG: 47 GR; 18 in mandible, 29 in maxilla TG: 59 GR; 20 in mandible, 39 in maxilla	NR	KW: CTG > CM
Schimitt et al., 2016 [37]	CG: CTG (n = 21) TG: XCM (n = 27) CAF	Mucograft^®^	Mandible (anterior region)	During 2° surgery	KW: FGG > CM
Thoma et al., 2016 [12]	CG: CTG (n = 10) TG: XCM (n = 10) Sutured grafts on periosteum without periodontal dressing	Fibro-Gide^® a^	Maxilla and mandible PM to PM	After implant placement	GT: CTG < CM
Cairo et al., 2017 [38]	CG: CTG (n = 30) TG: XCM (n = 30) CAT	Mucograft^®^	Maxilla and mandible	During 2° surgery	KW: CTG > XCM GT: CTG > XCM *
Zeltner et al., 2017 [39]	CG: CTG (n = 10) TG: XCM (n = 10) Sutured grafts on periosteum without periodontal dressing	Fibro-Gide^®^	Maxilla and mandible PM to PM	After implant placement	GT: CTG < CM
Huber et al., 2018 [19]	CG: CTG (n = 10) TG: XCM (n = 9) Sutured grafts on periosteum without periodontal dressing	Fibro-Gide^®^	Maxilla and mandible PM to PM	After implant placement	GT: CTG < CM
Pietruska et al., 2018 [28]	CG: CTG (n = 20) TG: XCM (n = 20) MCAT	Mucoderm^®^	Maxilla and mandible	NR	KW: CTG > CM * GT: CTG > CM *
Puzio et al.,2018 [16]	CG: CTG (n = 15) TG: XCM (n = 15) CAT	Mucograft^®^	Maxilla and mandible (anterior region)	3 months after implantation	GT: CTG > CM *
Nahas et al., 2019 [40]	CG: CTG (n = 15) TG: XCM (n = 15) MCAT	Mucograft^®^	Maxilla and mandible 82 GR CG: 40 TG: 42	NR	KW: CTG > CM
Vellis et al.,2019 [31]	CG: CTG (n = 30) TG: XCM (n = 30) Sutured grafts on periosteum without periodontal dressing	Mucograft^®^	Maxilla and mandible (posterior region)	After crown placement	KW: FGG > CM
Rakasevic et al., 2020 [41]	CG: CTG (n = 20) TG: XCM (n = 20)MCAT	Mucoderm^®^	Maxilla and mandible (114 multiple maxillary and mandibular type GR)	NR	KW: CTG < CM GT: CTG < CM *
Schmitt et al., 2020 [42]	CG: CTG (n = 17) TG: m CM (n = 17)	Mucoderm^®^	NR	During 2° surgery	GT: CTG > CM
Tarasenko et al., 2020 [29]	CG: FGG (n = 21) TG: CM (n = 19) CAF	Mucograft^®^	Mandible	During 2° surgery	KW: FGG > CM *
Thoma et al., 2020 [43]	CG: CTG (n = 10) TG: XCM (n = 8) Sutured grafts on periosteum without periodontal dressing	Fibro-Gide^®^	Maxilla and mandible PM to PM	After implant placement	GT: CTG > CM

STA: soft tissue augmentation, CG: control group, CTG: connective tissue graft, TG: test group, XCM: xenogeneic collagen matrix, CM: collagen matrix, CAT: coronally advanced tunnel, KMW: keratinized mucosa width, GT: gingival thickness, CAF: coronally advanced flap, mCM: monolayer collagen matrix, GR: gingival recession, NR: non-reported, ^a^ Geistlich Pharma AG, Wolhusen, Switzerland, ^b^ Botiss biomaterials GmbH, Zossen, Germany, * statistically significant.

## Data Availability

The data presented in this study are available on request from the corresponding author.
